# P-1702. Impact of Facility-level Rates of Lower Respiratory Cultures on Rates of Methicillin-Resistant Staphylococcus aureus and Pseudomonas aeruginosa among U.S. Hospitals, 2019-2023

**DOI:** 10.1093/ofid/ofaf695.1874

**Published:** 2026-01-11

**Authors:** Mia E Ferretti, Natalie McCarthy, Joseph D Lutgring, Hannah Wolford, Sujan Reddy, Scott Fridkin

**Affiliations:** Centers for Disease Control and Prevention, San Antonio, Texas; Centers for Disease Control and Prevention, San Antonio, Texas; Division of Healthcare Quality Promotion, Centers for Disease Control and Prevention, Atlanta, GA; CDC, Atlanta, Georgia; CDC, Atlanta, Georgia; emory university, Atlanta, Georgia

## Abstract

**Background:**

Understanding variability in culturing practices across facilities can inform the interpretation of surveillance data (e.g., comparing rates of pathogens across facilities). Diagnostic stewardship efforts aim to affect culturing practices. We explored facility-level variability of lower respiratory cultures (LRC) and associations between overall LRC rate and its association with facility-level rates of respiratory methicillin-resistant *Staphylococcus aureus* (MRSA) and *Pseudomonas aeruginosa* (PA).

**Figure:**
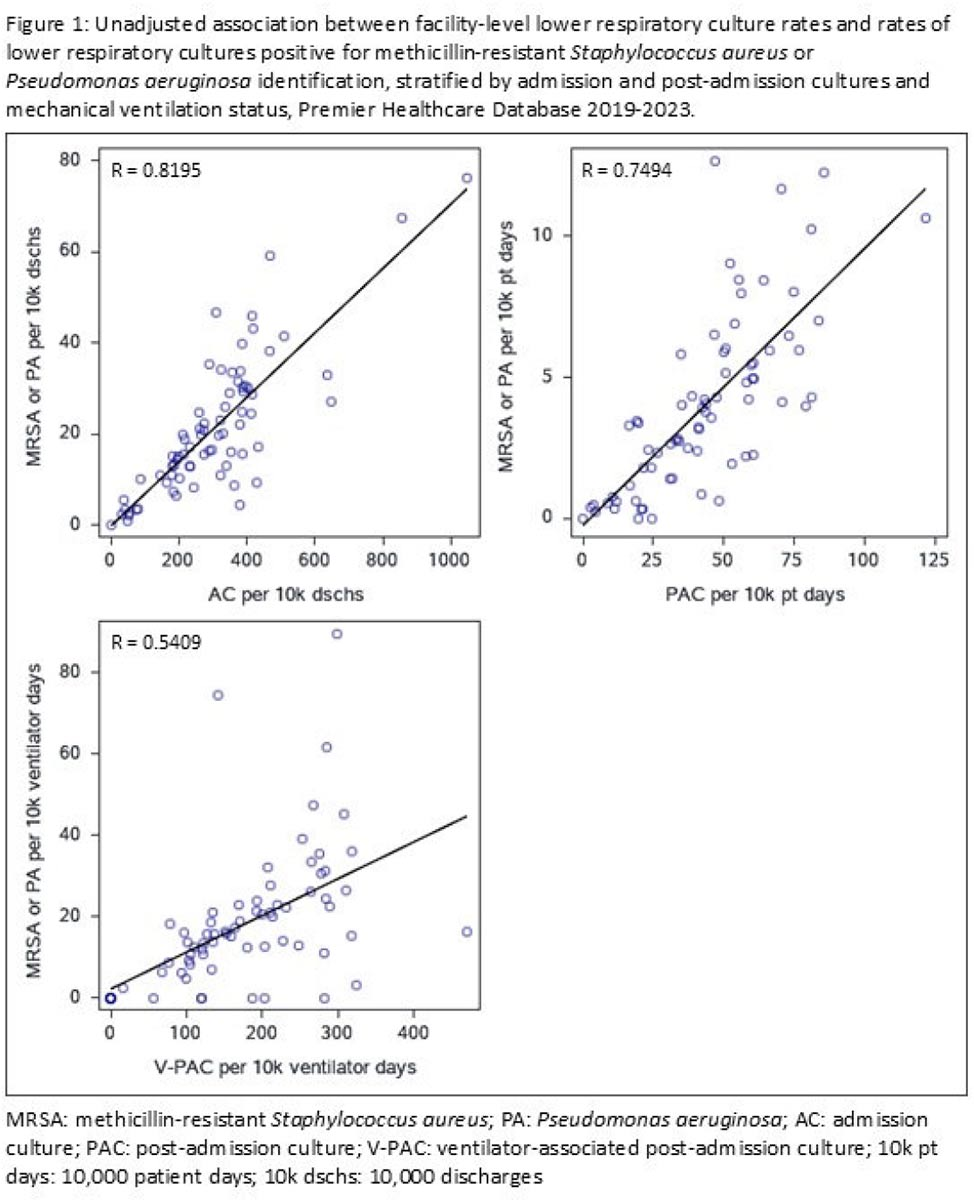

**Methods:**

Hospitalization and culture data from 2019-2023 were obtained from the Premier Healthcare Database. Facilities were included that reported hospitalization and LRC data every month of the study. Overall, LRC and LRC positive for MRSA or PA were categorized as admission cultures (AC), post-AC (PAC), and ventilator-associated PAC (V-PAC). ACs were collected within 3 days of admission, and PACs on or after day 4. V-PACs were PACs with a hospital charge for mechanical ventilation on the same day. Rates were reported as culture number per 10,000 discharges (AC), 10,000 patient days (PAC), and 10,000 ventilator days (V-PAC). We used multivariable negative binomial regression to assess the association of overall facility LRC rates and rates of LRC positive for MRSA or PA, adjusted for facility teaching status, urbanicity, size, region, year, and average length of stay.

**Results:**

Seventy-three facilities contributed 273,901 LRCs over the study period. LRC rates varied across facilities, with a median AC rate of 287.4 per 10,000 discharges (interquartile range [IQR] 172.8-397), median PAC rate of 42.4 per 10,000 patient days (IQR 23.8-59.8) and median V-PAC rate of 155.2 per 10,000 ventilator days (IQR 85.7-253.2). Unadjusted analyses (Figure 1) and multivariable models showed increased rates of LRC positive for MRSA or PA as overall LRC rates increased (p< 0.0001 for AC, PAC, and V-PAC).

**Conclusion:**

We observed considerable variability in facility LRC rates. Higher overall LRC rates were significantly associated with increased LRC pathogen detection. Information on diagnostic testing in facilities may provide important context for public health. Facility diagnostic stewardship will likely impact the clinical management of patients and surveillance of selected pathogens.

**Disclosures:**

All Authors: No reported disclosures

